# Tigers on the Move: The Impact of Climate Change on Tiger Distribution in Nepal

**DOI:** 10.1002/ece3.72397

**Published:** 2025-11-02

**Authors:** Ajay Karki, Kelly H. Dunning, Saroj Panthi, Kathan Bandyopadhyay, Abhinaya Pathak, Saneer Lamichhane, Abdul Ansari, Shiva Pariyar, Shambhu Paudel, Sarita Lama, Krita K. C., Shyam Kumar Shah, John L. Koprowski

**Affiliations:** ^1^ Department of Zoology and Physiology, Haub School of Environment and Natural Resources University of Wyoming Laramie Wyoming USA; ^2^ Department of National Parks and Wildlife Conservation Kathmandu Nepal; ^3^ Ministry of Forest and Environment Gandaki Nepal; ^4^ Department of Ecology, Behavior and Evolution, School of Biological Sciences University of California San Diego California USA; ^5^ Department of Wildlife Ecology and Conservation, School of Natural Resources and Environment University of Florida Gainesville Florida USA; ^6^ University of Canterbury Christchurch New Zealand; ^7^ Texas Park and Wildlife Department Austin Texas USA; ^8^ Ministry of Forests and Environment Bagmati Nepal; ^9^ Kathmandu Forestry College Kathmandu Nepal

**Keywords:** bioclimatic variables, coexistence, future habitat overlap, MaxEnt, SSPs

## Abstract

The Bengal tiger ( *Panthera tigris tigris* ), a flagship and umbrella species of the South Asian forest ecosystem, has declined dramatically in population and geographic distribution due to human‐caused habitat fragmentation and poaching over the past century. Global tiger populations may persist in the next century only if the size and quality of the current habitat remain unchanged. Our first‐of‐its‐kind study in Nepal assesses whether these habitat requirements are in place through an analysis of habitat suitability to predict the future habitat of tigers in varying climatic scenarios across the country. We collected tiger‐presence location (GPS points) from tiger surveys conducted by the Department of National Parks and Wildlife Conservation, Nepal, in 2018 and 2022 across the country. We used MaxEnt software in varying Shared Socio‐economic Pathways (SSP 245 and 585) employing eight bioclimatic and two topographic variables to predict the future habitats of the tiger in 2050, 2070, and 2090. In the SSP 245 scenario, tiger habitat could increase for all three time periods, but in the SSP 585 scenario, the habitat will increase only in 2050. Interestingly, in both scenarios, tiger habitat will increase by more than 80% in 2050. The expanded habitat in all scenarios is outside of protected areas and northeast of the current habitat. This indicates that extreme climate change scenarios with more industrialization, urbanization, and land use change have a greater impact on tiger habitat. Furthermore, tiger habitat qualitatively shifts from protected areas to outside protected areas in the human‐dominated landscape. This creates more challenges for conservationists and managers as human‐tiger interaction may surge. Proactive management solutions to protect Nepal's tigers for the next century could include expanding or establishing new protected areas, establishing connectivity and corridors between the tiger habitats, in addition to anticipatory efforts to address human‐wildlife conflicts that will emerge in this changing landscape.

## Graphical abstract

The impact of climate on tigers' habitat in Nepal in 2050, 2070, and 2090. The results indicates that tiger habitat will shifts outside protected area in future.

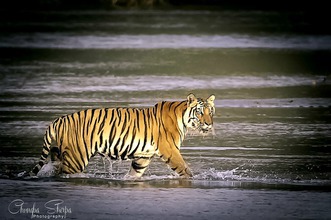

## 1 Introduction

Climate change has emerged as a significant threat to global biodiversity conservation (Heller and Zavaleta 2009; Howden et al. 2003; Tian et al. 2014; Watson et al. 2012), and ecosystems and species assemblages have responded to these patterns (IPCC 2018; Bellard et al. 2012; Walther et al. 2002). Human societies have acclimated to a stable climate, but as climate changes, possible impacts could occur to our water systems, biodiversity, and ecosystems, and could potentially disrupt the intricate relationships between flora, fauna, and their environment (Bates et al. 2008). Extensive documentation exists on the impact of climate change on the range shifts of species, including both contraction and expansion (Parmesan 2006; Schwartz et al. 2006; Thomas et al. 2004; Verboom et al. 2010; Walther et al. 2002; Wasserman et al. 2012). Species may respond to climate change by adapting to the evolving environment, altering their distribution ranges, or facing extinction (Holt 1990; Wiens et al. 2009). For example, shifts in marine species (Erauskin‐Extramiana et al. 2020; Hazen et al. 2013; VanDerWal et al. 2013) demonstrate how species shift towards the poles to minimize climate change impact. Endangered species facing climate change either must shift their range to climates within their tolerance limits or adapt locally through genetic changes resulting in altered behavior; failing to do so may result in global or local extinction (Fuller et al. 2010; Mitchell et al. 2008; Romero‐Mujalli et al. 2021). The situation is more crucial for large mammals such as tigers ( *Panthera tigris* ) that require large areas; range shifting options in fixed boundary protected areas in human‐dominated landscapes are unlikely options, and the rate of climate change is too fast relative to generation time for genetic adaptation to occur (Hetem et al. 2014).

The Bengal tiger, henceforth tiger, is a flagship and umbrella species (Barua 2011), which is listed as an endangered species on the IUCN Red List of Threatened Species (Goodrich et al. 2022) and under Appendix I of CITES (CITES 2025). Tigers are one of the most threatened big cats, with a population that has declined dramatically in the last century (Miquelle et al. 2015). Climate change is an overarching stressor to the global tiger population recovery (GTRP 2023). Due to the impacts of climate change, the tiger population may only persist over the next century if the size and quality of existing habitat patches remain unaltered in the face of climate change (Tian et al. 2014). For example, the tiger population in far eastern Russia and northeastern China is projected to decline and face the highest extinction risk in the next century under some climate change scenarios, despite the potential expansion of suitable habitat northward (Tian et al. 2014). Similarly, a 28 cm increase in the mean sea level from the year 2000 would result in a staggering 96% reduction of tiger habitat in Bangladesh's Sundarbans, with the number of breeding individuals dwindling to < 20 (Loucks et al. 2010; Upadhayaya and Baral 2020). Currently, there are 114 (89–146) tigers in the Bangladesh Sundarbans (Aziz et al. 2019) and most tiger habitats are < 1 m above sea level (Canonizado and Hossain 1998). Climate change disrupts ecosystems and habitats, potentially causing species migration and altering natural food webs, which may lead to tigers venturing closer to human settlements, resulting in conflicts that often result in retaliatory killings of tigers, and attacks on humans and livestock (Rahim et al. 2015; Treves and Karanth 2003). Hence, conservation efforts in the face of climate change should address current challenges and anticipate and adapt to future climate conditions (Glick et al. 2011).

Climate change has been identified as one of the major challenges to tiger conservation in Nepal (DNPWC 2023). The climate has been changing in Nepal faster than the global average (Upadhayaya and Baral 2020) and is expected to become warmer and drier in the coming decades (WBG 2022). These changes will most likely affect the distribution of the habitat and population of the Bengal tiger. The future distribution of various mammal species can be accurately predicted by correlating climatic factors alongside current species ranges and projecting future scenarios (Chichorro et al. 2019; Hetem et al. 2014). Predicting suitable habitats and creating detailed maps of landscape features has become increasingly crucial for monitoring changing conditions and evaluating the capacity of landscapes to support native mammal species (Bhandari et al. 2022).

During this research, we explored how tigers may respond to potential radiative forcing across various Shared Socio‐Economic Pathways (SSPs). The SSP scenarios outline future global conditions based on key social, economic, and technological development changes (Lehtonen et al. 2021; Meinshausen et al. 2020). These scenarios include a range of shifts in demographics, economics, geopolitics, institutions, sociological variables, and technological advances—providing insights into how societal changes could impact responses to climate change threats (Frame et al. 2018). We have used MaxEnt software to predict the future habitat for the years 2050, 2070, and 2090 under two SSPs (245 and 585). SSP 245, also called the business‐as‐usual scenario, describes a status quo scenario in which economic, social, and technological patterns follow historical trends. Technology is assumed to advance gradually together with moderate economic growth. SSP 585, also called the fossil‐fuel development scenario, describes a future with development predominantly driven by fossil fuel dependence. Education, technological advancement, and urbanization rates are high in this scenario (Sanderson et al. 2019). We choose to use SSPs rather than Representative Concentration Pathways (RCPs) because RCPs cannot define socio‐economic characteristics with difficulty in mapping societal changes, such as population dynamics, education levels, and governmental policies, with climate goals like limiting global warming to below 2°C. SSPs aim to overcome this by outlining how societal choices can lead to changes in radiative forcing by the end of the century (Meinshausen et al. 2020). Further, SSPs are seen as the most relevant input to employ when examining the impacts of human advancements and the adoption of greenhouse gas emission control policies (Meinshausen et al. 2020).

This study aims to investigate and forecast the potential habitat suitability and distribution patterns of tigers using a MaxEnt approach under changing climatic scenarios in Nepal. MaxEnt only requires species presence data and works efficiently even with a low number of species presence data (Khan and Khalid 2021). Because of its robustness and accuracy, this is one of the most popular approaches for predicting the possible distribution of the species (Phillips and Dudík 2008) under different climate change scenarios (Karki and Panthi 2021; Sharma et al. 2020; Zhao et al. 2021). The study is the first of its kind in Nepal to predict the future habitat of tigers in varying climatic scenarios across the country. We seek to address the following research questions: (1) What bioclimatic and topographical variables significantly influence the geographic distribution of tigers in different climate change scenarios? (2) Which geographical areas are ecologically and environmentally suitable for tigers under current and projected future climate change scenarios? (3) Will the ecological range of tigers diminish or shift under the anticipated future climatic conditions? The outcomes will not only aid in identifying and mapping core geographical areas with habitat suitability for tigers but could also assist in delineating potential areas that require protection in order to conserve tiger habitats and promote the coexistence of local communities' dependent on these ecosystems and tigers.

## 2 Data and Methods

### 2.1 Study Area

**FIGURE 1:**
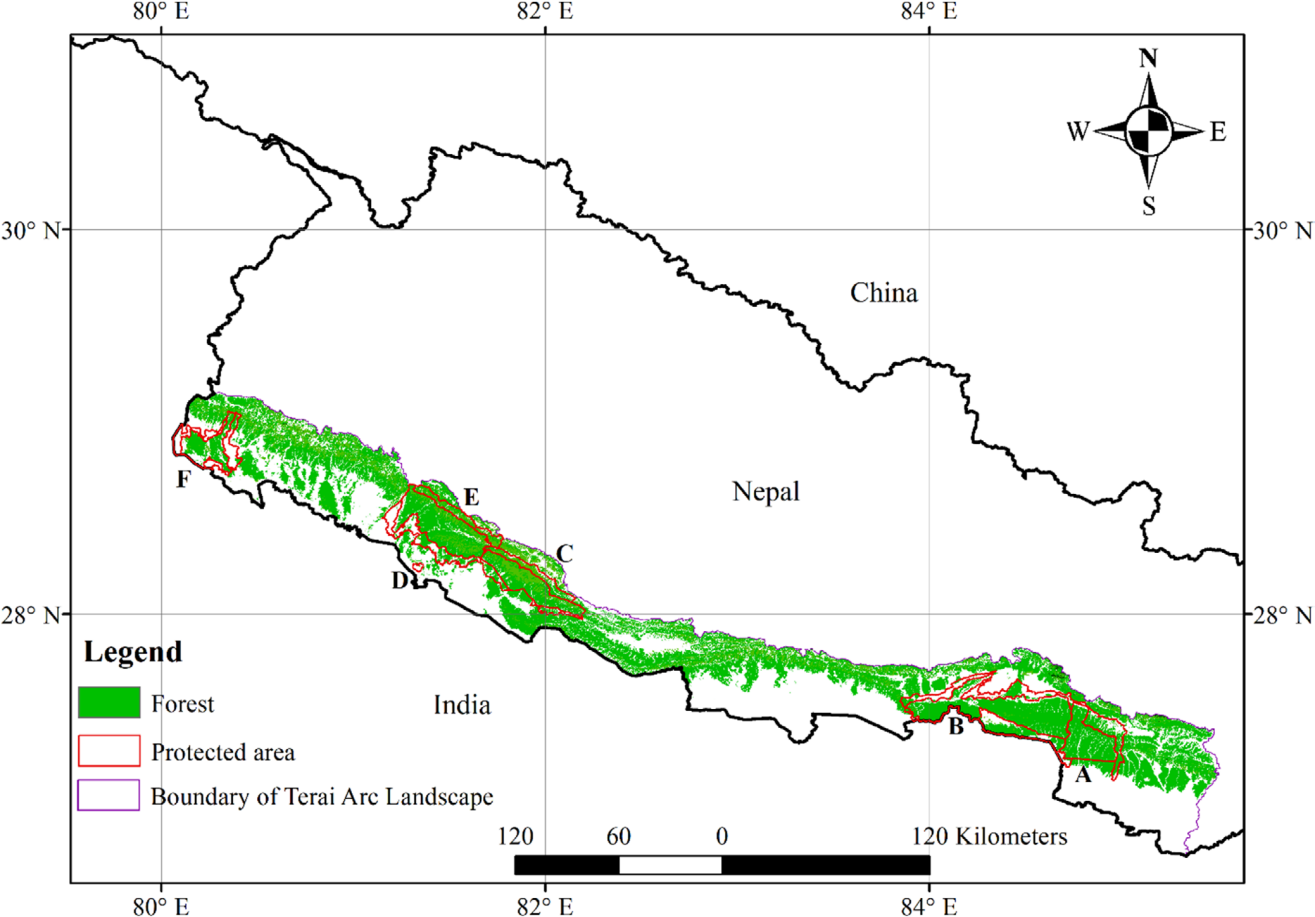
Major habitats and land management protections for tigers ( *Panthera tigris* ) across the study area in Nepal. Tiger habitat in Nepal is stretched along the border of India; this area has major corridors and bottlenecks that connect the tiger's habitat in India and Nepal. A, B, C, E, and F are Parsa, Chitwan, Banke, Bardia, and Shuklaphanta National Parks, and D is Khata Corridor.

Our study was conducted across the Nepal landscape where tigers have been reported and censused (Figure 1). We focused on the Terai Arc Landscape (TAL) of Nepal. The TAL in Nepal spreads across 19 districts, including five tiger‐bearing protected areas, namely, Parsa, Chitwan, Banke, Bardia, and Shukla Phanta National Parks. These parks lie along the international border of India and are connected through many north–south corridors that stretch between the two countries. The TAL in Nepal covers 2.4 million hectares, and more than half of the area (1.32 million ha with > 10% crown cover forest) is covered by forests (FRA 2015). The TAL has highly productive grasslands and riverine forests that support Asia's largest herbivores and carnivores (MoFE 2015). The fauna in this area includes 85 species of mammals, 565 species of birds, 47 species of herpetofauna, and more than 125 species of fish (MoFE 2015). Among them, the Asian elephant ( *Elephas maximus* ), Gangetic dolphin ( *Platanista gangetica* ), greater one‐horned rhinoceros ( *Rhinoceros unicornis* ), and tiger ( *Panthera tigris* ) are key faunal species. A recent survey shows 355 tigers share this landscape (DNPWC and DFSC 2022) with a population of more than nine million (CBS 2023) who are mostly dependent on forests for their livelihood (Pandey et al. 2021).

### 2.2 Data Collection

#### 2.2.1 Occurrence Points of the Tiger

The GPS locations of the tigers were obtained from nationwide systematic camera trapping tiger surveys conducted by the government of Nepal in 2018 and 2022 (DNPWC and DFSC 2022). Altogether, we obtained 3994 occurrence points. For analysis, we filtered and selected only one point within a 1 km × 1 km grid cell to minimize the spatial autocorrelation and to ensure that each presence point is unique. Ultimately, we used 1437 unique occurrence points for the final analysis.

#### 2.2.2 Bioclimatic and Topographical Data

Bioclimatic data were obtained from the Worldclim open‐access database (www.worldclim.com), and topographic data were accessed from USGS GTOPO30. Altogether, 10 variables were used in our analysis; the remaining 12 variables were removed from the final analysis because of their collinearity (Table 1).

**TABLE 1:** Bioclimatic and topographic variables used in preliminary and final modeling of the potential tiger habitat in Nepal and their attributes.

Categories	Variables	Abbreviations	Unit	Included or not in final model	Source of data
Bioclimatic (version 2)	Annual mean temperature	Bio1	°C	No	Worldclim
	Mean diurnal range (mean of monthly (max‐min temp))	Bio2	°C	Yes	
	Isothermality (Bio2/Bio7)	Bio3	Dimensionless	No	
	Temperature seasonality (SD)	Bio4	°C	No	
	Maximum temperature of the warmest month	Bio5	°C	No	
	Minimum temperature of the coldest month	Bio6	°C	No	
	Temp. Annual range (bio5‐bio6)	Bio7	°C	No	
	Mean temperature of the wettest quarter	Bio8	°C	Yes	
	Mean temperature of the driest quarter	Bio9	°C	Yes	
	Mean temperature of the warmest quarter	Bio10	°C	No	
	Mean temperature of the coldest quarter	Bio11	°C	No	
	Annual precipitation	Bio12	Mm	No	
	Precipitation of the wettest month	Bio13	Mm	Yes	
	Precipitation of the driest month	Bio14	Mm	Yes	
	Precipitation seasonality (coefficient of variation)	Bio15	Dimensionless	Yes	
	Precipitation of the warmest quarter	Bio16	Mm	No	
	Precipitation of the coldest quarter	Bio17	Mm	Yes	
	Precipitation of the wettest quarter	Bio18	Mm	Yes	
	Precipitation of the driest quarter	Bio19	Mm	No	
Topographic	Elevation	Elevation	M	No	USGS GTOPO 30
	Aspect	Aspect	Degree	Yes	
	Slope	Slope	Degree	Yes	

### 2.3 Data Analysis

We initially used 19 bioclimatic variables and three topographic high‐resolution variables (2 km × 2 km) for modeling under two SSP scenarios (245 and 585) to predict the potential habitat range of the tiger in 2050, 2070, and 2090. The bioclimatic data used for the prediction of 2050 represent the mean value from 2041 to 2060; similarly, those used for 2070 represent the mean value from 2061 to 2080, and those used for 2090 represent the mean value from 2081 to 2100. Further, the values of bioclimatic variables for our study area were extracted by sampling a prediction raster in ArcGIS (version 10.3). The collinearity between the variables was tested using R software (PresenceAbsence) (R Core team 2024). Highly correlated variables (VIF > 10) were removed from further analysis to reduce multicollinearity and obtain the model's reliability. After removing the autocorrelated variables, only 10 variables (Table 1) were considered for further analysis. We assumed that topographic variables would not change during the simulation period, 2050, 2070, and 2090. Data from the Model for Interdisciplinary Research on Climate (MIROC6) Global Climate Model (GCM) were used as recommended for interdisciplinary research on climate change (Watanabe et al. 2010). Future projections for 2050, 2070, and 2090 were derived from IPSL‐CM6A‐LR and MIROC6 models for different SSPs (245 and 585), respectively. We trained the model using 70% of the data (presence locations), reserving the remaining 30% for model validation purposes (Karki and Panthi 2021; Sharma et al. 2020). The model was configured with 10 replications, 10,000 maximum iterations, and 1000 background points (Barbet‐Massin et al. 2012). Additionally, we conducted a Jackknife test to assess the individual contributions of bioclimatic and topographic variables to the model (Pearson et al. 2007).

**FIGURE 2:**
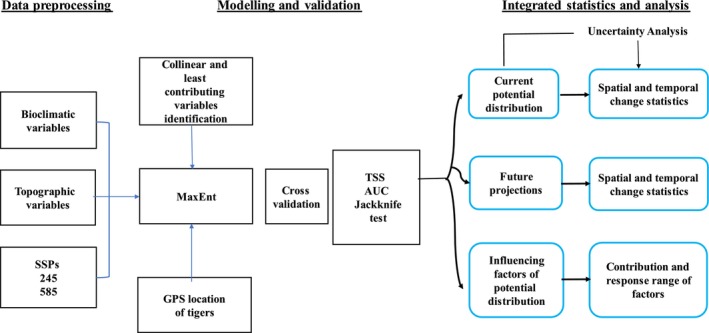
Systematic flowchart of the methodology employed in predicting the future distribution of tigers in Nepal. The first column with three boxes explains bioclimatic and topographic variables that were used in two SSPs, 245 and 585. The second, third, and fourth columns, together with six boxes, explain how we input the GPS points into the MaxEnt model, checked for the variable's collinearity and contribution towards the model, cross‐validated the output, and checked for TSS, AUC, and the Jackknife test. The last block of boxes explains how the current and future distribution of tigers is determined, and which factors are most influential in determining this distribution.

We evaluated model accuracy using both threshold‐dependent and threshold‐independent methods. In the threshold‐independent approach, accuracy was assessed by calculating the area under the curve (AUC) of the model. A higher AUC indicates higher model performance, with AUC > 0.9 indicating excellent performance, 0.7–0.9 signifying moderate performance, and AUC < 0.7 suggesting poor model performance (Barbet‐Massin et al. 2012; Elith et al. 2006). Although AUC is widely used, it has been criticized for being influenced by geographical extents (Pearce and Ferrier 2000; Pearson et al. 2007). To address this limitation, we used the true skill statistics (TSS) index as a threshold‐dependent method, optimizing sensitivity and specificity with a provided threshold (Barbet‐Massin et al. 2012; Elith et al. 2006; Phillips et al. 2006). TSS values range from −1 to +1, with values closer to −1 indicating near‐random model performance and a value of +1 indicating a perfect fit of the model (Allouche et al. 2006). Maximizing TSS with a threshold is recommended (Allouche et al. 2006; Liu et al. 2013). We utilized a threshold to convert the continuous probability map into a binary map, delineating suitable and unsuitable habitat areas. Subsequently, we compared the geographical distribution maps generated under SSPs 245 and 585 for 2050, 2070, and 2090 with the current and prospective distribution maps to assess changes in tiger habitat suitability over time. Our methodological overview (Figure 2) is adapted from Ali et al. (2023).

## 3 Results

### 3.1 Model Performance and Variable Significance

**FIGURE 3:**
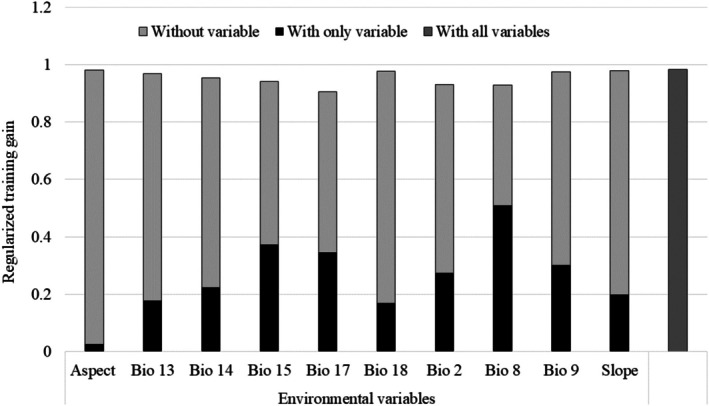
Jackknife test of regularized training gain of the 10 most influential variables and their importance identified in predicting the future habitat of tigers in Nepal.

The AUC (0.87) and TSS (0.7) values of the model represent the “fit” and “predictive reliability” of the model. The Jackknife testing demonstrates the significance of explanatory variables in prediction models. The wettest quarter (bio8) was highly rated as a predictor variable based on regularized training gain in the current climate scenario (Figure 3). The regularized training gain measures how much better the model's distribution aligns with the presence data compared to the uniform distribution. The term “without variable” refers to the impact of removing that particular variable from the model, whereas the term “with only variable” refers to the model's performance when only that specific variable is used. The term “with all variables” refers to the model's result when all variables are used in the training process.

Using the Jackknife method for the AUC, we can assess the importance of the variable only (blue bar), compared to when the variable is removed (light green bar: Figure 3). All explanatory variables contributed to the improvements of the model. A plausible explanation is the removal of the highly collinear and least influential 12 variables from the model. The ranking of importance (without variable) depicts that the bio17 variable (precipitation of the coldest quarter) is the leading variable, followed by bio8 (mean temperature of the wettest quarter), bio2 (mean diurnal range of temperature), and bio15 (precipitation seasonality) variables. This means that there would be the highest decrease in the training gain while removing the bio17 variable from the model. Similarly, the permutation importance of bio17 variables is highest among the variables used for the modeling (Table 2).

**TABLE 2:** Permutation importance of the variables used in models for the modeling of tiger response.

S. No.	Variables	Permutation importance
1	Precipitation of the coldest quarter (bio 17)	26
2	Precipitation seasonality (coefficient of variation) (bio 15)	21.8
3	Mean diurnal range (mean of monthly (max‐min temp)) (bio 2)	16.9
4	Precipitation of the driest month (bio 14)	12.6
5	Mean temperature of the wettest quarter (bio 8)	12.3
6	Mean temperature of the driest quarter (bio 9)	3.9
7	Precipitation of the wettest quarter (bio 18)	2.8
8	Precipitation of the wettest month (bio 13)	2.6
9	Slope	0.9
10	Aspect	0.2

TSS values obtained are between 0.66 and 0.71, and AUC scores between 0.86 and 0.88 (Table 2); TSS values > 0.6 and AUC values > 0.8 are considered to be excellent (Komac et al. 2016).

### 3.2 Variable Response Curves

**FIGURE 4:**
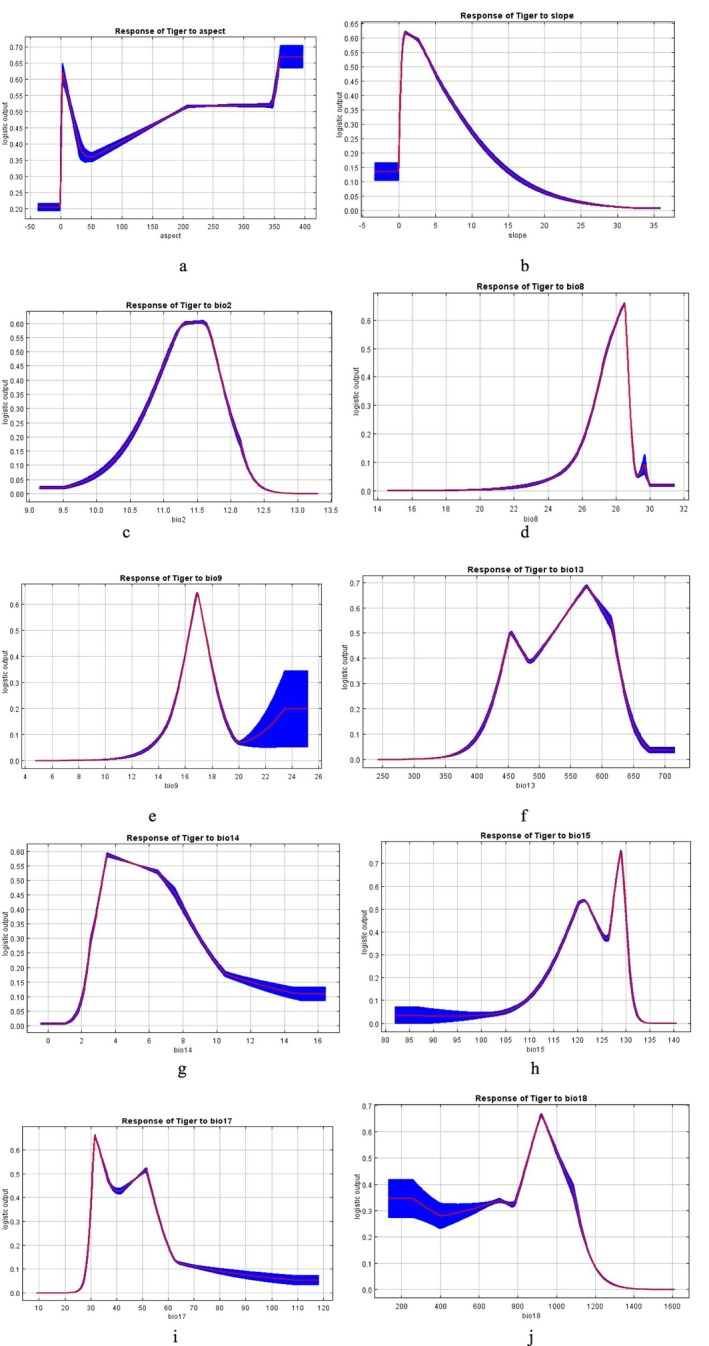
MaxEnt model response curves of the most important ten variables determining habitat suitability of Nepal's tigers. Fig 4a‐ aspect; 4b‐ slope; 4c‐ bio 2 or mean diurnal range (mean of monthly max‐min temperature); 4d‐ bio 8 or mean temperature of the wettest quarter; 4e‐ bio 9 or mean temperature of the driest quarter; 4f‐ bio 13 or precipitation of the wettest month; 4g‐ bio 14 or precipitation of the driest month; 4h‐ bio 15 or precipitation seasonality (coefficient of variation); 4i‐ bio 17 or precipitation of the coldest quarter; 4j‐ bio 18 or precipitation of the wettest quarter.

The Figure 4a – aspect (degree); Figure 4b – slope (degree); Figure 4c – bio 2 (°C) or mean diurnal range (mean of monthly max–min temperature); Figure 4d – bio 8 (°C) or mean temperature of the wettest quarter; Figure 4e – bio 9 (°C) or mean temperature of the driest quarter; Figure 4f – bio 13 (mm) or precipitation of the wettest month; Figure 4g – bio 14 (mm) or precipitation of the driest month; Figure 4h – bio 15 (dimensionless) or precipitation seasonality (coefficient of variation); Figure 4i – bio 17 (mm) or precipitation of the coldest quarter; Figure 4j – bio 18 (mm) or precipitation of the wettest quarter are the model response curves for the most important variables. The x‐axis of Figure 4a–j represents the variable value for different variables, and the y‐axis represents the projected habitat of the tiger for the respective variable (Figure 4). For example, according to the model response curves, the 25–50 mm precipitation of the coldest quarter (bio17) is the most favorable for tiger distribution (Figure 4i). Additionally, 10°C–11.6°C of the mean diurnal temperature range (bio2) is the most favorable for tiger distribution (Figure 4c).

### 3.3 Current Habitat and Habitat Projection

**FIGURE 5:**
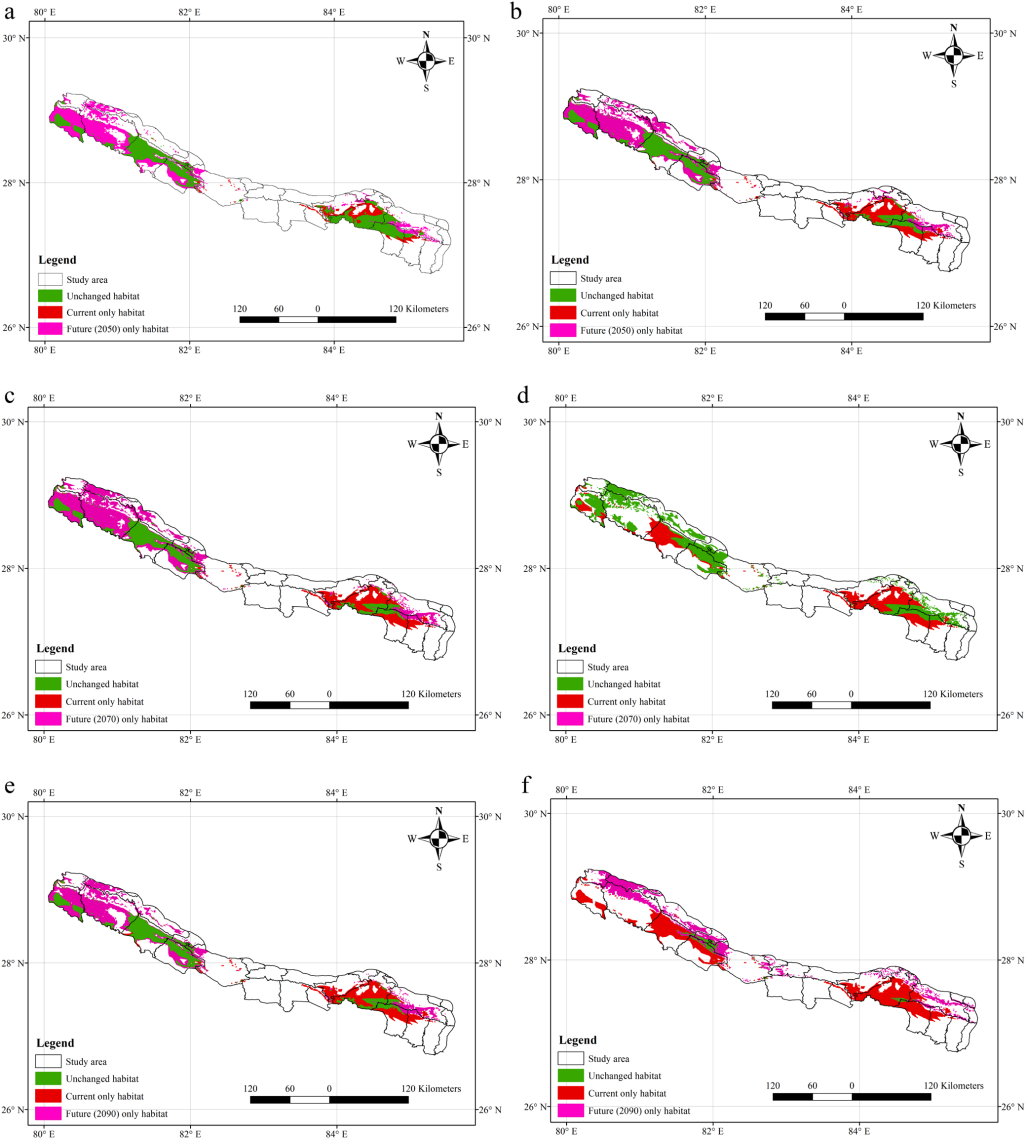
Future habitat distribution scenarios for tigers in Nepal under 245 (left) and 585 (right) SSPs for 2050 (a, b), 2070 (c, d) and 2090 (e, f). The green area is the unchanged habitat, the pink area is the future habitat or potential habitat, and the red area is the current only habitat and is predicted to disappear in the future.

We configured six models based on two projected future climate change scenarios (SSPs 245 and 585) and three time periods (2050, 2070, and 2090) to understand the suitability of tigers on a temporal basis (Figure 5). The current habitat of tigers across Nepal is 5625 km^2^, which is 3.8% of the country's total land area (Table 3). Our analysis indicates that most of the current tiger habitat is inside protected areas (64%, 3623 km^2^), whereas 36% (2002 km^2^) of the habitat extends beyond the protected areas.

**TABLE 3:** Current and future tiger habitat for the years 2050, 2070, and 2090 in two climate change scenarios (SSPs 245 and 585). Currently, the 2002 km^2^ habitat is outside the protected areas and is predicted to increase in both scenarios for all three time periods.

Habitat categories	Area of PA	Current habitat	Future habitat in km^2^ in different scenarios					
			SSP 245			SSP 585		
			2050	2070	2090	2050	2070	2090
Protected areas (PA)	5528	3623	4133	3437	3229	3503	2006	820
Outside PAs	0	2002	5094	6112	4785	5392	3416	3013
Total	5528	5625	9227	9549	8014	8895	5422	3833

Our analysis shows that in 2050, the habitat will be increased by 64% and 58% in SSPs 245 and 585 scenarios, respectively. In 2070, the habitat is predicted to increase by 70% in the SSP 245 scenario, and the habitat is predicted to decrease by 10% in the SSP 585 scenario as compared to the current scenario. If we look at 2090, the tiger habitat would be increased by 42% in the SSP 245 scenario, and the habitat would be decreased by 38% in the SSP 585 scenario.

Our analysis indicates that SSP 245 predicts an increase in the habitat of tigers in Nepal, and the SSP 585 scenario initially helps increase the habitat but has gradually started decreasing after 2060. Regardless of the climate scenarios deployed, the outside‐PA habitat is always greater than the inside‐PA habitat for all 3 time periods.

## 4 Discussion

### 4.1 SSPs and the Scenarios

The current distribution of tigers is influenced by habitat availability and quality, prey abundance and density, and anthropogenic pressure such as deforestation, poaching, and urban area expansion. Tigers require large, continuous forests with sufficient prey to sustain their populations and avoid areas with high human disturbance (Karanth and Stith 1999; Sunquist and Sunquist 2002). Conservation efforts, including protected areas and wildlife corridors, play a critical role in maintaining and connecting tiger populations across fragmented landscapes (Dinerstein et al. 2007). Additionally, small and isolated populations face genetic challenges, which can limit long‐term survival and further restrict their distribution (Goodrich et al. 2022). We focused on tiger habitat under two future climate scenarios (SSP 245 and 585). In the SSP 585 scenario, tiger habitat is predicted to decrease in 2070 and 2090. Since SSP 585 is considered more extreme, it will bring about a more abrupt habitat change than that of SSP 245. Climate change has diverse impacts, and habitat loss caused by climate change has a significant role in driving a wide variety of species toward extinction (Akay et al. 2011; Dadashi‐Jourdehi et al. 2020; Inkley et al. 2004). Similarly, future range contraction for tigers, lions, and leopards has been reported in other studies (Ebrahimi et al. 2021; Kina et al. 2020; Loveridge et al. 2022; Mitchell et al. 2024). Similar to our results, the rapid decline in the tiger population and suitable tiger habitats by 2050 and a complete loss of this species by 2070 is predicted for Bangladesh (Mukul et al. 2019). In northern Thailand, approximately 39% of current tiger habitats will shift by 2050 as a result of the combined effect of land use and climate change (Trisurat et al. 2015). Realized suitable habitat fragments—areas currently supporting tiger populations—are more critical for conservation than areas identified as potentially suitable but unoccupied, particularly given the strong negative impact of human density and disturbance on habitat quality (Tian et al. 2014). This underscores the importance of preserving existing undisturbed habitats, such as those in the Sundarbans region of Bangladesh, where climate change is projected to exacerbate human‐tiger conflict by transforming these minimally human‐dominated landscapes into more heavily human‐impacted areas (Rahim et al. 2015).

The future of any wildlife species will principally depend on their ability to tolerate a wide range of climatic conditions, such as changes in temperature, limited or heavy rainfall, etc. (Alamgir et al. 2015; Feeley and Silman 2010; Ramirez‐Villegas et al. 2014). In this study, five of the eight bioclimatic variables incorporated into the model were identified as important, all of which were derived from precipitation‐related factors. Nevertheless, tigers are known to occupy a remarkably broad ecological niche, ranging from sea level to elevations of approximately 4500 masl, and spanning diverse habitat types, including tropical rainforests, subtropical forests, and dry grasslands (Goodrich et al. 2022). Therefore, actual distribution losses could be less than predicted because tigers have high adaptive capacity and can live under a wide range of climatic and habitat conditions and can utilize various types of prey (Kawanishi and Sunquist 2004). The changes in forest cover—the primary determinant of tiger habitat—are influenced more strongly by socio‐economic pressures on land use and political decisions regarding forest retention than by precipitation‐related variables. However, our scenarios (SSPs) do not incorporate enough socio‐economic variables and political variables, nor do they account for potential future trends in human pressures on forests within the study area. This limitation may constrain the accuracy and predictability of our habitat projections. Additionally, robust conservation efforts are being made by the government of Nepal and other stakeholders that may help maintain the tigers' range. The tiger prey base has increased with the interventions made to adapt to climate change in potential climate refugia for tigers in Nepal (Advani 2023). However, range loss might also be higher than predicted because tigers may not occupy their full range due to prey abundance, competition with other carnivores, interaction with humans, and dispersal limitations. These variables influence the future distribution of tigers differently across regions. For instance, the human population is higher in the eastern part of Nepal (CBS 2023), and prey density is highest in the far‐west and low‐lying regions in Banke National Park of Nepal (DNPWC and DFSC 2022).

### 4.2 Habitat Modeling and Interaction With People

Habitat loss due to the cascading effects of climate change has led to an increased risk of extinction for many species (Hotta et al. 2019; Rezaei et al. 2022; Segan et al. 2016; Thuiller et al. 2006; Urban 2015). The impact of climate change on the future distribution of species is well known. The available habitat of carnivores will be decreased continuously in the future due to climate change (Bhandari et al. 2022; Farrington and Li 2024; Jones et al. 2016; Suel et al. 2018; Wilmers and Getz 2005; Zahoor et al. 2021). Additionally, changes in the distribution pattern of prey species may have negative effects on predators, especially on specialist predators, because decreased spatial overlap scenarios between prey and predators could lead the predators towards extinction (Farrington and Li 2024; Jones et al. 2016; Suel et al. 2018; Zahoor et al. 2021). To tackle these impacts and challenges, adaptation by the animals and incorporation of management regimes such as establishing new protected areas, corridors between habitats, expansion of existing protected areas, and establishing buffer zones are needed (Araújo et al. 2011; Di Minin et al. 2016; Mazaris et al. 2013; Psaralexi et al. 2017; Rezaei et al. 2022).

Most tiger habitats in Nepal will be outside protected areas in the future, and the current habitat will be shifted towards the north‐east, except in 2070 under 585 scenarios. The projected future expansion of tiger habitat under varying scenarios is expected to overlap with current habitat by as little as 6% and—under more optimistic conditions—up to 93%. However, in that best‐case scenario, as much as 55% of habitat within protected areas is still projected to be lost. Meanwhile, the currently occupied habitat outside of protected areas is already under significant anthropogenic pressure—and with ongoing development trends, such pressures are expected to intensify in the coming years (Neupane 2024). A recent study highlights this increasing threat, noting that unprotected areas face increasing pressures from human expansion, which leads to increased habitat fragmentation and jeopardizes critical ecological corridors even beyond protected area boundaries (Bhattarai and Kindlmann 2018; Carter et al. 2022). This creates further challenges for tiger conservation in Nepal in the days to come, because no tigers were recently documented in the mountains of the Churia range of Kapilvastu, Palpa, and Rupandehi districts of Nepal (Subedi et al. 2021). Similarly, a tiger was reported at an altitude of 2511 masl in Dadeldhura district of Nepal (Thapa et al. 2022); another tiger was recorded at an altitude of 3165 masl in Ilam district of eastern Nepal (Bista et al. 2021). Tigers were also recorded at 3602 and 3274 masl in India, and 4028 masl in Bhutan (Shrestha et al. 2021), suggesting that high elevations might be considered as potential future habitat for tigers. We have not included such occasional presence outliers of tigers in this study because no additional evidence of tiger residency and reproduction has been recorded from these sites. Tiger presence in the Churia range sheds light on hope for the connection of the eastern and western populations of Nepal via this range (Subedi et al. 2021) and indicates that higher altitude habitats may work as climate refugia for the lowland tiger population of western Nepal (Thapa et al. 2022). A major problem in the high altitude of Nepal is the low abundance of the prey base, so this might be the limiting factor for the tiger's distribution in other areas except the lowlands of Nepal.

Furthermore, tigers avoid areas of human dominance (Bhattarai and Kindlmann 2013; Carter et al. 2023), but our study shows that tigers' habitat will overlap and they will interact with humans and anthropogenic activities in the future, which may exacerbate the human–tiger conflict in the future. However, human–tiger interaction is not only determined by population sizes and their overlap but also by the choices people make about conservation (Sanderson et al. 2019). In 2022, Nepal nearly tripled its wild tiger population, having pledged at the Global Tiger Summit in Russia in 2010 to double the population by that year (DNPWC and DFSC 2022). However, human–tiger conflicts are rising, and at least 19 tigers are in captivity for attacking people (Pers. Comm 2024). Human–tiger conflict is already a major conservation challenge in habitats close to human settlements and is expected to increase to create further conservation challenges (Kina et al. 2020). The rate of human casualties in Nepal outside protected areas increased by 925% between 1998 and 2006, as compared to 1979 and 1988, where only a 289% increase was reported inside the protected areas during the same period (Gurung et al. 2008). Similarly, human–tiger conflict was 850% more frequent in the buffer zone of Chitwan National Park of Nepal from 2007 to 2016 as compared to inside the National Park (Lamichhane et al. 2017). Our findings indicate that the conflict between humans and tigers will increase considerably in human‐dominated landscapes as compared to forest‐dominated landscapes and protected areas because tiger habitat will increase outside of most areas in the future. If intensive habitat management activities are not performed and human–tiger conflict reduction actions are not implemented, it might be difficult to conserve the tiger population sustainably in the future in these areas. This study did not investigate the potential connectivity between predicted future tiger habitats in Nepal and current tiger habitats in India, nor between the projected habitats in the Churia region within Nepal. Nonetheless, Bhatt et al. (2023) identified nine critical corridors and associated pinch points that facilitate wildlife corridors between Nepal's existing tiger habitats, transboundary habitats in India, and the remaining intact forests of the Churia. The conservation of these intact habitats, along with the management of corridors and pinch points, is therefore needed for sustaining metapopulation connectivity and ensuring long‐term genetic exchange. To ensure evidence‐based conservation planning, future research should integrate projections of climate change impacts with corridor suitability mapping. Such an approach would yield a more robust framework for safeguarding landscape connectivity and enhancing the resilience of tiger populations under dynamic environmental and anthropogenic pressures.

### 4.3 Models' Strength and Accuracy

Climate changes are expected to affect the magnitude and frequency of climate extremes in the future (Butt et al. 2016), which may exacerbate other biodiversity‐related threats as well (Brook et al. 2008; Ockendon et al. 2014). While climate models have effectively projected the distribution on a large scale (Guisan and Zimmermann 2000; Rahbek and Graves 2001), some researchers have raised concerns about the lack of detailed information on species interactions and dispersal processes. Studies have highlighted the importance of considering human‐related factors in addition to climate data when assessing species distribution (Davis et al. 1998; Iverson et al. 1999; McCarty 2001). Artificial Neural Networks (ANN) were used to analyze the impact of human‐related factors on species distribution and found that these factors did not significantly influence projections (Thuiller et al. 2004); however, incorporating human‐related factors could enhance the accuracy of modeling results (Pearson et al. 2007). While climate change alone may contribute to the extinction of certain species, a combination of climatic and anthropogenic effects is likely responsible for the extinction of others (Lorenzen et al. 2011). Considering only climate‐related variables in species distribution modeling may lead to an overestimation or underestimation of future suitable habitats (Mukul et al. 2019). The SSP scenarios integrated into our model have considered future economic growth, demographic changes, environmental degradation, and developmental factors to enhance the accuracy of species distribution prediction. Model predictions can be enhanced by incorporating dispersal and dispersal pathways (Araújo et al. 2011), yet predicting these remains challenging (Elith and Leathwick 2009). This study indicates that tiger habitat is projected to expand in the future under most scenarios; however, much of this expansion is expected to occur outside of current protected areas. It is important to note that our model did not account for several critical ecological and anthropogenic factors, including prey availability and distribution, prey density, forest crown density, habitat quality, and daily human pressures such as forest product extraction. Consequently, predictions of habitat shift outside PAs and increases in forest cover, derived from a limited set of variables, may not fully predict the complexity of future habitat dynamics. Prey abundance is a key determinant of tiger density (Karanth et al. 2004), yet it was not incorporated into our model. Tigers may occupy a broader range of habitats than a single prey species, and we did not model the potential future distribution of the prey base. Under future climate change scenarios, prey species distributions may be more strongly constrained than those of tigers, potentially forcing tigers to move outside protected areas in search of food. Such movements could, in turn, increase the likelihood of human–tiger conflicts. Further, our model does not include the human–tiger conflict as a predictor, but we know that it is a major conservation challenge (Bodasing 2022; Fernández‐Sepúlveda and Martín 2022; Johnson et al. 2023; Ripple and Beschta 2012) and will be a key factor in determining future tiger habitat. Additionally, human perception and acceptance are important factors to be considered for the successful recovery of large carnivores and their range expansion (Drouilly and O'Riain 2021). A key limitation of MaxEnt is its reliance on presence‐only data, which makes predictions sensitive to sampling bias and background selection (Elith et al. 2011; Merow et al. 2013). The model also assumes niche conservatism, thereby limiting its reliability under novel climate conditions (Araújo and Peterson 2012). Furthermore, MaxEnt does not explicitly account for biotic interactions, dispersal constraints, or dynamic land‐use changes, which are critical under climate change scenarios (Guisan and Thuiller 2005). Additionally, slope and aspect were included as predictor variables in our model; however, they did not emerge as important determinants of tiger habitat suitability within Nepal. This outcome is likely attributable to the relatively uniform habitat conditions across much of Nepal's tiger range. Nonetheless, tigers are known to occur at elevations up to 4500 masl in other range countries and inhabit diverse terrains with varying slopes (Goodrich et al. 2022). In such contexts, the findings of our model may have limited applicability.

## 5 Conclusion and Conservation Implications

This is the first nationwide study in Nepal to predict tiger habitat considering current and future climate change scenarios (SSPs 245 and 585) for 2050, 2070, and 2090. In most of the scenarios, the current habitat is predicted to shift towards the north‐east of the current habitat, and most of the predicted habitat is outside the existing protected areas of Nepal. This necessitates planned habitat expansion to cover major tiger habitats and corridors of connectivity, which ultimately can help reduce the conflict with humans, provided that the government involves local stakeholders in planning and designing conservation works and management actions (Kina et al. 2020).

Considering the inevitable impacts of climate change on global ecosystems and species (Pant et al. 2021), managers and policymakers must prioritize tiger conservation efforts. This can be achieved by expanding designated areas for tiger conservation and establishing corridors to facilitate dispersal and transboundary movements of tigers. Habitat restoration, connecting fragmented habitats, and reducing disturbance in tiger habitats are key ways to promote human–tiger co‐existence. Tigers need a larger home range (DNPWC 2023; Majumder et al. 2012), and conservation activities need to be implemented over larger areas to conserve this species, which will have a significant cost and require substantial resources. Our research can help inform managers to optimally allocate the limited resources for maximum conservation impact. Additionally, future conservation strategies should consider the potential impacts of climate change on tigers and their ability to adapt, the avoidance of human‐dominated landscapes to expand the tiger habitat, and the co‐existence of humans and tigers in human‐dominated landscapes. It is recommended that future studies integrate additional ecological and anthropogenic factors, such as land‐use changes, prey abundance and density, the presence of other carnivores, and poaching data for tigers and their prey, into habitat modeling. While our research is based solely on tiger presence data from Nepal, it is important to note that tiger habitats are transboundary and connected to India through established wildlife corridors. Consequently, modeling habitat across the entire Terai Arc Landscape, incorporating these additional factors, would likely yield more comprehensive and reliable insights to guide effective conservation planning.

## Author Contributions

**Ajay Karki:** conceptualization (equal), data curation (equal), formal analysis (equal), funding acquisition (equal), investigation (equal), methodology (equal), project administration (equal), resources (equal), software (equal), supervision (equal), validation (equal), visualization (equal), writing – original draft (equal), writing – review and editing (equal). **Kelly H. Dunning:** conceptualization (equal), formal analysis (equal), writing – original draft (equal), writing – review and editing (equal). **Saroj Panthi:** conceptualization (equal), data curation (equal), formal analysis (equal), methodology (equal), software (equal), writing – original draft (equal). **Kathan Bandyopadhyay:** methodology (equal), software (equal), writing – review and editing (equal). **Abhinaya Pathak:** conceptualization (equal), data curation (equal), methodology (equal), writing – original draft (equal), writing – review and editing (equal). **Saneer Lamichhane:** conceptualization (equal), formal analysis (equal), methodology (equal), software (equal), writing – original draft (equal), writing – review and editing (equal). **Abdul Ansari:** writing – review and editing (equal). **Shiva Pariyar:** conceptualization (equal), methodology (equal), software (equal), writing – original draft (equal), writing – review and editing (equal). **Shambhu Paudel:** conceptualization (equal), data curation (equal), formal analysis (equal), methodology (equal), writing – original draft (equal), writing – review and editing (equal). **Sarita Lama:** conceptualization (equal), formal analysis (equal), methodology (equal), software (equal), writing – original draft (equal), writing – review and editing (equal). **Krita K. C.:** conceptualization (equal), data curation (equal), formal analysis (equal), methodology (equal), software (equal), writing – original draft (equal), writing – review and editing (equal). **Shyam Kumar Shah:** data curation (equal), formal analysis (equal), methodology (equal), software (equal), writing – original draft (equal), writing – review and editing (equal). **John L. Koprowski:** conceptualization (equal), data curation (equal), formal analysis (equal), investigation (equal), methodology (equal), resources (equal), software (equal), supervision (equal), validation (equal), visualization (equal), writing – original draft (equal), writing – review and editing (equal).

## Conflicts of Interest

The authors declare no conflicts of interest.

## Supporting information


**Appendix S1:** ece372397‐sup‐0001‐AppendixS1.csv.

## Data Availability

The authors confirm that all the required data is uploaded as Supporting Information S1.
